# Selective ablation of TRA-1-60^+^ pluripotent stem cells suppresses tumor growth of prostate cancer

**DOI:** 10.7150/thno.78915

**Published:** 2023-03-27

**Authors:** Jordan M. White, Nicholas Ramos, Allen-Dexter Saliganan, Joon-Yong Chung, Meghan Bell, Jacob Lindquist, Kayla Conner, Wendy N. Wiesend, Michael Schopperle, Steve M. Patrick, Seongho Kim, Elisabeth I. Heath, Freddy E. Escorcia, Nerissa T. Viola

**Affiliations:** 1 Department of Oncology, Karmanos Cancer Institute, Detroit, MI 48201; 2 Cancer Biology Graduate Program, Wayne State University, School of Medicine, Detroit, MI 48201; 3 Molecular Imaging Branch, Radiation Oncology Branch, National Cancer Institute, National Institutes of Health, Bethesda, MD 20892; 4 Department of Anatomic Pathology, Beaumont Health System, Royal Oak, MI 48073; 5 Curemeta, LLC, Boston, MA 02210

**Keywords:** TRA-1-60, prostate cancer stem cells, immunoPET imaging, Lutetium-177, radionuclide therapy

## Abstract

**Purpose:** TRA-1-60 (TRA) is an established transcription factor of embryonic signaling and a well-known marker of pluripotency. It has been implicated in tumorigenesis and metastases, is not expressed in differentiated cells, which makes it an appealing biomarker for immunopositron emission tomography (immunoPET) imaging and radiopharmaceutical therapy (RPT). Herein, we explored the clinical implications of TRA in prostate cancer (PCa), examined the potential of TRA-targeted PET to specifically image TRA^+^ cancer stem cells (CSCs) and assessed response to the selective ablation of PCa CSCs using TRA-targeted RPT.

**Experimental Design:** First, we assessed the relationship between TRA (*PODXL*) copy number alterations (CNA) and survival using publicly available patient databases. The anti-TRA antibody, Bstrongomab, was radiolabeled with Zr-89 or Lu-177 for immunoPET imaging and RPT in PCa xenografts. Radiosensitive tissues were collected to assess radiotoxicity while excised tumors were examined for pathologic treatment response.

**Results:** Patients with tumors having high *PODXL* CNA exhibited poorer progression-free survival than those with low *PODXL*, suggesting that it plays an important role in tumor aggressiveness. TRA-targeted immunoPET imaging specifically imaged CSCs in DU-145 xenografts. Tumors treated with TRA RPT exhibited delayed growth and decreased proliferative activity, marked by Ki-67 immunohistochemistry. Aside from minor weight loss in select animals, no significant signs of radiotoxicity were observed in the kidneys or livers.

**Conclusions:** We successfully demonstrated the clinical significance of TRA expression in human PCa, engineered and tested radiotheranostic agents to image and treat TRA^+^ prostate CSCs. Ablation of TRA^+^ CSCs blunted PCa growth. Future studies combining CSC ablation with standard treatment will be explored to achieve durable responses.

## Introduction

Tumor heterogeneity adversely affects treatment outcomes for patients with cancer. The cancer stem cell (CSC) model is one of the main explanations for intratumoral heterogeneity 1. CSCs comprise a small population of a diverse mix of cell phenotypes that co-inhabit the tumor mass 2. Their innate ability to self-renew and reprogram is posited to be a driving force for tumor initiation, progression and eventually, metastasis 3. The reprogramming hallmark of CSCs is characterized by cellular plasticity arising from the activation of transcription factors associated with germ cells and embryonic stem cells 4,5. Metastatic cancer cells exhibit a more dedifferentiated phenotype that is congruent with their aggressive behavior 6. Prostate cancer (PCa) harbors heterogeneous cell phenotypes including CSCs 7,8. Biopsied samples from patients with prostate cancer, have confirmed the presence of several stemness markers including, but not limited to CD133, Oct-4, NANOG, and TRA-1-60 (TRA) 9,10. Additional reports have empirically proven that CSCs isolated from PCa are responsible for self-renewal and tumor initiation 11, and exhibit drug resistance 8,12. In patients with metastatic castration-resistant prostate cancer (mCRPC), limited treatment options are available, none of which are curative 13. Typically, patients are treated with docetaxel and later cabazitaxel. Recently, [^177^Lu]Lu-PSMA-617, the first prostate-specific membrane antigen (PSMA)-selective radiopharmaceutical therapy (RPT) was approved for patients with disease progression following first-line taxane treatment 14. The findings demonstrated an absolute overall survival benefit of approximately ~ 4 months compared with protocol-defined standard-of-care 15. Importantly, while survival was prolonged, cures remain elusive, likely resulting in part from tumor heterogeneity (e.g., tumor cells that do not express PSMA), and treatment-resistant CSCs 3. Thus, while challenging, precise targeting and eradication of CSCs represent a promising strategy that has the potential to dramatically alter the outcomes of patients with PCa as well as other malignancies.

Because cell signaling pathways associated with embryonic stem cells are often co-opted in oncogenesis, they have become emerging targets for cancer therapy 16. TRA-1-60 (TRA) has long been recognized as an embryonic stem cell (ESC) marker 17. TRA is a high-molecular weight proteoglycan that is localized on the surface of the transmembrane protein podocalyxin (*PODXL*) 18. As a signature transcription factor of ESC, TRA expression leads to a highly proliferative status with associated pluripotency 4,19. TRA has been implicated in the invasiveness, metastatic potential, and treatment relapse of a number of cancers including prostate, oral squamous cell, ovarian, gastrointestinal and pancreatic cancers, as well as follicular lymphoma and germ cell tumors 10,20-26. It is reportedly cleaved off podocalyxin and secreted into the blood in many cancer phenotypes including testicular, pancreatic and prostate cancers 24,27,28. Moreover, its nominal expression in normal and differentiated tissues further underscores its appeal as a therapeutic and diagnostic cancer target 18. In prostate cancer, Rajasekhar *et al.* demonstrated the tumor-initiating potential of TRA^+^ PCa cells 29. Our group has demonstrated that high expression of TRA in high-grade PCa resulted in metastatic disease 10. Similarly, another study that compared primary and metastatic prostate lesions identified a TRA^+^ cell population as specific to metastases 28. Interestingly, in one patient, the presence of a TRA^+^ population within the primary lesion predicted the development of metastatic disease with four months lead time over conventional detection methods. Because the presence of TRA^+^ cells is associated with aggressive disease, non-invasive detection of these cell populations can help guide prognosis and treatment interventions. While molecular biology approaches can be utilized to detect TRA^+^ cells from liquid biopsies, immunopositron emission tomography (immunoPET) can identify and quantify these cells in space and time, which can be leveraged for personalized interventions (e.g. escalation of local treatment). Furthermore, confirmation of TRA expression may predict for response to TRA-targeted therapy, which can thwart tumor progression.

PSMA-targeted RPT represents a new second line therapy for patients with mCRPC. Still, many exhibit low PSMA expression identified via PET imaging, and will not achieve the clinical responses of those with disease with high PSMA expression. Even for patients who initially have high PSMA expression and achieve overall survival benefits, few, if any, are cured. Accordingly, many groups have and continue to evaluate the combination of PSMA-based RPT and other systemic therapies, including immune checkpoint blockade, and inhibitors of DNA damage repair 30-32. To the best of our knowledge, CSC targeting is yet an unexplored approach.

Given the critical role of CSCs in tumor recurrence, and the specificity of TRA for CSCs, we aimed to develop a TRA-specific agent that can be used to identify, and later ablate prostate cancer CSCs. Herein, we first analyzed The Cancer Genome Atlas (TCGA) and SU2C/PCF databases to determine whether the expression of *PODXL*, the gene which encodes for TRA, was implicated in patient outcomes. We then report our radiotheranostic approach, whereby we first imaged, and then treated TRA^+^ CSC populations in a preclinical model of metastatic prostate cancer using minimal PSMA-expressing DU-145 cell-line derived xenografts. Previously, we showed that our TRA-specific immunoPET agent [^89^Zr]Zr-DFO-Bstrongomab (Bsg) localized to TRA^+^ cells in *in vivo* tumor models 33. In this study, we leveraged this immunoPET agent to detect TRA^+^ cells in PCa xenografts and inform our development of a TRA-selective RPT agent using lutetium-177 (β^-^, t_1/2_ ~ 6.67 d), a beta-emitting radioisotope. Because bone marrow, liver, and kidney toxicities are common with RPT, we prospectively monitored blood markers for related end-organ injury during treatment. The histopathology of the liver and kidneys was examined to assess radiation-induced tissue damage as well. Tumor proliferative activity was examined via Ki-67 immunohistochemistry at the end of the therapy study to assess anti-tumor effect of treatment.

## Materials and Methods

### Correlation of progression-free survival with *PODXL* expression in prostate cancer patients

The Cancer Genome Atlas (TCGA) PanCancer data for primary prostate cancer (PRAD dataset) were downloaded from cBioPortal (http://www.cbioportal.org) to investigate progression-free survival (PFS). Copy number alteration (CNA) was used for the expression analysis of *PODXL,* the gene which encodes TRA. For metastatic prostate adenocarcinoma, the CNA expression levels of *PODXL* were obtained from SU2C/PCF Dream Team metastatic prostate adenocarcinoma data using libraries prepared by the polyA method via cBioPortal 34. The normalized Z-scores relative to all samples were used to determine expression levels. The Kaplan-Meier (KM) methods were used to generate the survival curves, and a log-rank test was carried out to compare between groups. To determine the associations of *PODXL* with PFS, the CNA expression levels of *PODXL* were dichotomized into two groups (high vs. low) by the median.

### Cell Culture and Xenografts

All cell lines (ATCC) were grown in 5 % CO_2_ in a sterile humidified environment at 37 ºC in media. Metastatic PCa-derived DU-145 were grown in the RPMI-1640 and EMEM, respectively**.** Both media were supplemented with 10 % FBS and 1 % Penicillin/Streptomycin. All cell lines were routinely tested for mycoplasma using a mycoplasma detection kit (Lonza). Authenticity was certified by the Biobanking and Correlative Services Core at Wayne State University.

Animal experiments were conducted in accordance with the guidelines established by the Wayne State University Institutional Animal Care and Use Committee (IACUC) and the Division of Laboratory Animal Resources (DLAR). DU-145 and PC-3 prostate cancer cells were purchased from ATCC while 22rv1 was generously provided by Dr. Jason Lewis (MSKCC). All cell lines (5×10^6^ cells) were injected subcutaneously on the shoulder of 6-8 week old male athymic nude mice (Envigo RMS) in 150-200 μL 1:1 media:Matrigel (Corning, 354428). Tumor dimensions were monitored with calipers 2-3× weekly. Tumor volumes were calculated using the ellipsoid formula: length × width × height × π/6. Mice with tumor volumes of 100-200 mm^3^ were used in this study.

### Cell Sorting and Re-implantation

Cells from DU-145 xenografts were isolated using a tissue dissociation kit (Miltenyi) and tissue dissociator (GentleMACs, Miltenyi) according to the manufacturer's protocol. Dissociated cells were incubated with either anti-TRA-1-60-AF488 (sc-21705, diluted 1:100, Santa Cruz) or anti-CD133-PE (TMP4, diluted 1:200, eBioscience) or combined. Following incubation, the cells were strained with a 35 µm nylon mesh strainer. After gating for viability, TRA-positive and TRA-negative cells were collected under sterile conditions using a Sony Biotechnology SY3200 cell sorter. The collected cells were washed, pelleted and resuspended in a 1:1 media:Matrigel. Both groups of cells (1,000 - 2,000 cells per mouse) were re-implanted subcutaneously in male SCID mice.

### Immunofluorescence staining for TRA-1-60, CD44 and CD133

Formalin-fixed paraffin-embedded DU-145 xenograft tissue sections were deparaffinized, rehydrated and subject to heat-induced antigen retrieval using antigen retrieval buffer (pH 6.0; Dako, Carpinteria, CA) in a pressure cooker. Sections were blocked with 2.5% normal horse serum for 30 min and incubated with mixture of rabbit monoclonal anti-CD133 antibodies (clone D2V8Q, diluted 1:100; Cell signaling, Danvers, MA) and mouse monoclonal anti-TRA (clone TRA-1-60 (S), diluted 1:1000; Cell signaling) or mixture of rabbit monoclonal anti-CD44 antibodies (clone E7K2Y, diluted 1:1000; Cell signaling) and mouse monoclonal anti-TRA (clone TRA-1-60 (S), diluted 1:1000; Cell signaling) for 1 h at room temperature, followed by incubation with mixture of Alexa Fluor 488 goat anti-rabbit IgG (ThermoFisher Scientific, Waltham, MA) and Alexa Fluor 555 goat anti-rabbit IgG (ThermoFisher Scientific). After nucleus visualization with DAPI, sections were mounted with Prolong Diamond antifade (ThermoFisher Scientific) and imaged on a Zeiss Axio Imager 2 (Carl Zeiss Microscopy, Jena, Germany).

### Synthesis of [^89^Zr]Zr-DFO-Bstrongomab

Conjugation of Bstrongomab (Bsg, Curemeta, LLC) and radiolabeling with Zr-89 have been previously published 33. Briefly, Bsg was conjugated with *p*-isothiocyanato-benzyl-desferrioxamine (DFO, Macrocyclics, Inc.) by adding 33 nmol of DFO (20 mM in DMSO) to 6.6 nmol of Bsg in saline, pH ~ 9. The reaction was incubated at 37 ºC for 1.5 h. Excess unbound DFO was removed by centrifugal filtration at 3,000 rpm for 10 min using a 30 kDa centrifugal filter (30 kDa MWCO, GE Vivaspin V-500). Purification was performed in triplicate using saline as the eluent. The pH of [^89^Zr]Zr-oxalate (74 MBq, 2 mCi, 3D Imaging, LLC) was adjusted to pH ~ 7.0 with 1 M Na_2_CO_3_ in metal-free water. The radiometal was added to the Bsg-DFO conjugate (0.4 mg, 2.67 nmol in saline) and incubated for 0.5 - 1.5 h at room temperature. The reaction was quenched by the addition of 5 μL of 50 mM of ethylenediaminetetraacetic acid (EDTA, pH ~7.0). Free, unbound ^89^Zr was removed by centrifugal filtration. Radiolabeling efficiency and purity were examined by radio-instant thin layer chromatography (radio-iTLC, Eckert & Ziegler) using silica gel-impregnated strips (Agilent Technologies) with 50 mM EDTA as the mobile phase.

### Synthesis of [^177^Lu]Lu-CHX-A”-DTPA-Bstrongomab

Bsg was conjugated to *p*-isothiocyanato-benzyl-cyclohexyl-diethylenetriamine pentaacetic acid (CHX-A”-DTPA, Macrocyclics, Inc.) in accordance with previously published protocols 35. CHX-A”-DTPA (33 nmol of 20 mM stock in dimethyl sulfoxide) was added to a solution of Bsg in 1× PBS, pH ~ 9 and incubated at 37 ºC for 1-1.5 h. Unconjugated CHX-A”-DTPA was removed by centrifugal filtration (30 kDa MWCO, GE Vivaspin 500). Purification was performed in triplicate. CHX-A”-DTPA-Bsg was buffer exchanged with 0.2 M ammonium acetate, pH ~ 5.5 prior to the addition of ^177^Lu. No carrier added [^177^Lu]Lu-chloride (462.5 MBq or 12 mCi, SpectronRx) was added to CHX-A”-DTPA-Bsg (~2.5 mg). After 1 h of incubation at room temperature, the reaction was quenched with 5 μL of 50 mM EDTA (pH ~7.0). The purity was tested via radio-iTLC similar to the protocol described above.

### PET Imaging

[^89^Zr]Zr-DFO-Bsg (7.4-9.25 MBq, 200-250 μCi) was administered intravenously (i.v.) via the lateral tail vein in DU-145 bearing mice. PET scans were acquired between 4-120 h post-injection (p.i.) using a Focus 220 microPET (Siemens Concorde Microsystems) or Albira Si PET/CT (Bruker) scanner. During imaging, the mice were anesthetized with 2% isoflurane (Henry Schein) in air. The PET images were reconstructed using filter-back projections. Volumes-of-interest (VOIs), for the tumor and select organs, were manually measured on serial planar sections with either ASIPro VM^TM^ software (version 6.3.3.0, Concorde Microsystems) or PMOD (PMOD Technologies, LLC.). The VOIs were expressed as % injected dose per milliliters of tissue (%ID/mL) and reported as the mean ± S.D.

### SPECT/CT and therapy studies

For therapy studies, groups of mice bearing DU-145 palpable tumors were i.v. injected on the lateral tail vein with either 18.5 MBq (500 μCi, n=4) or 37.7 MBq (750 μCi, n=5) of [^177^Lu]Lu-CHX-A”-DTPA-Bsg. The untreated groups (n=6) served as controls. An additional cohort of mice with DU-145 tumors was administered 250 μg of non-radiolabeled Bsg. The tumor volumes were monitored every 2-3 days for 72 days. The rodents were observed for visible adverse effects including weight loss. Changes in tumor volume were presented as the % change normalized to the volume on the day of radiopharmaceutical administration.

A separate group of DU-145 mice (n=6) given 37.7 MBq were imaged via Siemens Inveon SPECT/CT at 120 h p.i. Images were analyzed using the Inveon Research Workplace v. 4.1. The animals were euthanized after imaging. Blood, kidneys and liver samples were collected for clinical chemistry and tissue toxicity analysis.

### Tissue distribution of [^177^Lu]Lu-CHX-A”-DTPA-Bstrongomab

DU-145 tumor-bearing mice were intravenously administered 0.74-0.925 MBq (20-25 μCi) of [^177^Lu]Lu-CHX-A”-DTPA-Bsg. The animals were sacrificed at 120 h p.i. via CO_2_ asphyxiation. Select tissues including the tumor were excised and weighed. Bound radioactivity was measured using a gamma counter (Perkin Elmer Wizard^2^ 2480), decay-corrected to the time of injection and presented as the percentage of injected dose per gram (%ID/g) of tissue.

### Hematological analysis

The mice were sacrificed immediately after the last imaging scan. Blood was separately collected into ethylenediamine tetraacetic acid (EDTA)-lined and lithium heparin-lined collection tubes (Sarstedt, Inc., Newton, North Carolina) and, if not processed immediately after collection, kept overnight at 4 °C, and processed once acclimated to room temperature. Complete Blood Count (CBC) blood samples collected in EDTA-lined tubes were analyzed using the Vetscan HM5 (Zoetis Inc., Parsippany, NJ), which was calibrated using a synthetic whole blood standard (Zoetis Inc., Parsippany, NJ). Basic Metabolic Panel (BMP) blood samples were collected in heparin-lined tubes and analyzed using the Vetscan VS2 (Zoetis Inc., Parsippany, NJ, USA) with comprehensive diagnostic rotors.

### Immunohistochemistry

DU-145 tumor sections and de-identified tissues from metastatic PCa patients obtained from the KCI Biobanking Core were stained for TRA using previously established protocols 33. Mouse livers and kidneys were collected and fixed immediately in 4 % formaldehyde for 24 h, transferred and stored in 70% ethanol before paraffin-embedding. Tissue sections (5 μm thick) were mounted on glass slides with sections stained with hematoxylin and eosin (H&E). H&E-stained slides from the liver and kidney samples were examined with an optical microscope (Nikon Eclipse C*i* Series, Tokyo, Japan) for histologic signs of radiation-induced injury. DU-145 tumor tissues stained for Ki-67 (clone 8D5; Cell Signaling Technologies, Cat # 9449T) were scanned and images were obtained using a Leica APERIO CS2 scanner. Proliferative cells were analyzed using Leica Aperio ImageScope v. 12.3.3 and expressed as the mean percentage of positively stained cells obtained from at least three different regions of the tumor section.

### Statistical Analysis

Data were analyzed using Graphpad Prism version 9.1.0. and presented as the mean ± S.D. unless otherwise stated. Statistical significance (set at p < 0.05) was derived via non-parametric Mann-Whitney t-test unless otherwise stated. Ki67, blood chemistry and metabolic panel were analyzed via one-way ANOVA using multiple comparisons test.

## Results

### Expression of *PODXL* in Primary Prostate Cancer May Be Linked to PFS

**Figure 1:**
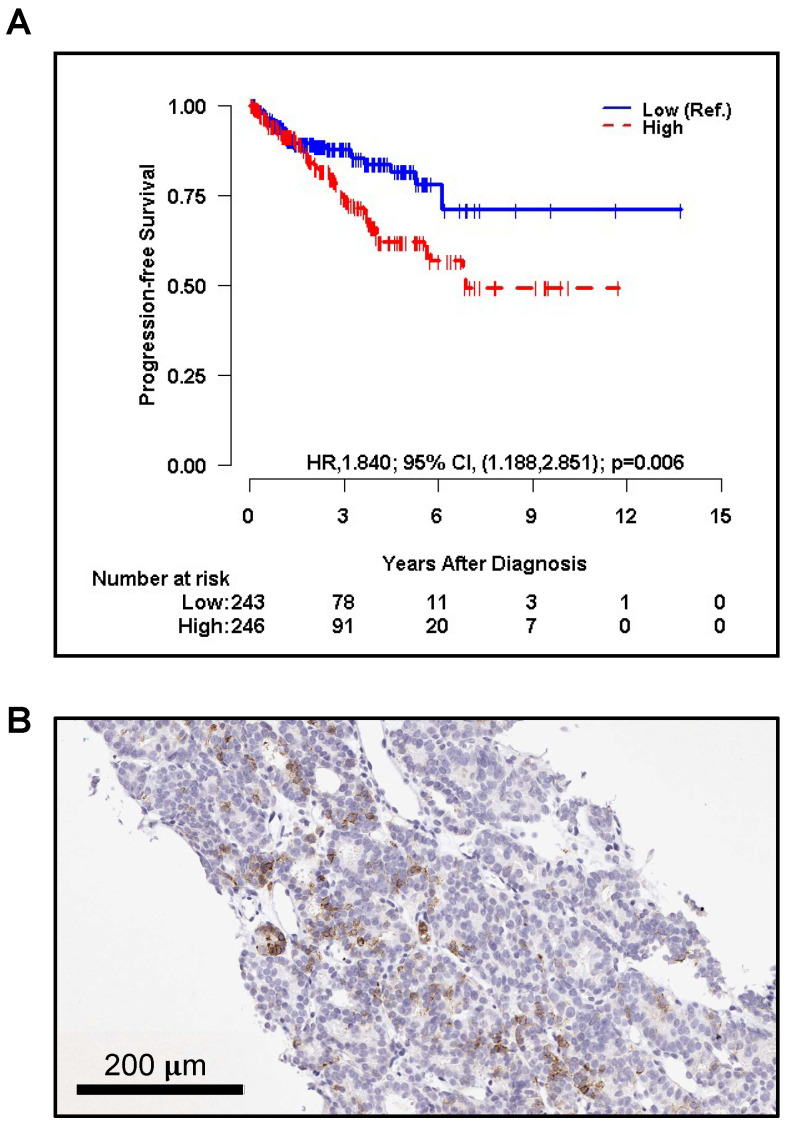
The Kaplan-Meier curve of **A.** progression-free survival (PFS) by copy number alterations (CNA) of *PODXL* (High vs. Low, Low as reference) of primary PCa patients respectively. CNA was dichotomized into two groups (High vs. Low) by the median for PFS. The p-value was obtained by a log-rank test. HR and CI stand for 'hazard ratio', and 'confidence interval', respectively.** C.** TRA^+^ cells (brown) are shown infiltrating lymph node tissue of a metastatic prostate cancer patient.

The association between the CNA expression level of *PODXL*, the gene encoding TRA, and progression-free survival (PFS) was assessed using the Kaplan-Meier (KM) curve and log-rank test using TCGA PanCancer primary prostate cancer data. The CNA level was dichotomized into two groups (high vs. low) by the median. The analysis revealed that patients with a high CNA of *PODXL* had significantly higher risk of progression or death than those with the low expression level (HR, 1.840; 95% CI, 1.188 to 2.851; p = 0.006) (**Figure 1A).** Interestingly, patients with larger tumor sizes (T2-T4) tended to have higher *PODXL* expression compared to patients with smaller tumors (T1) but this trend was not significant (**Figure S1A**).

SU2C/PCF Dream Team PCa data include outcomes from 444 mCRPC tumors but only 51 samples have both CNA levels and overall survival (OS) outcomes 34. These 51 mCRPC patients showed no significant association between *PODXL* and OS. (**Figure S1B**).

### TRA IHC identifies pluripotent cells within metastatic prostate cancer

Lymph node tissue was obtained from a patient with metastatic PCa (PSA score: 406 ng/mL) treated with radiation therapy post-androgen deprivation therapy and administered sipuleucel-T, abiraterone, docetaxel, cabazitaxel, and enzalutamide. Focal positive staining in ~5-10% of the tumor cells was displayed (**Figure 1B**).

### PET delineates TRA^+^ cells in cell-derived prostate cancer xenografts

**Figure 2:**
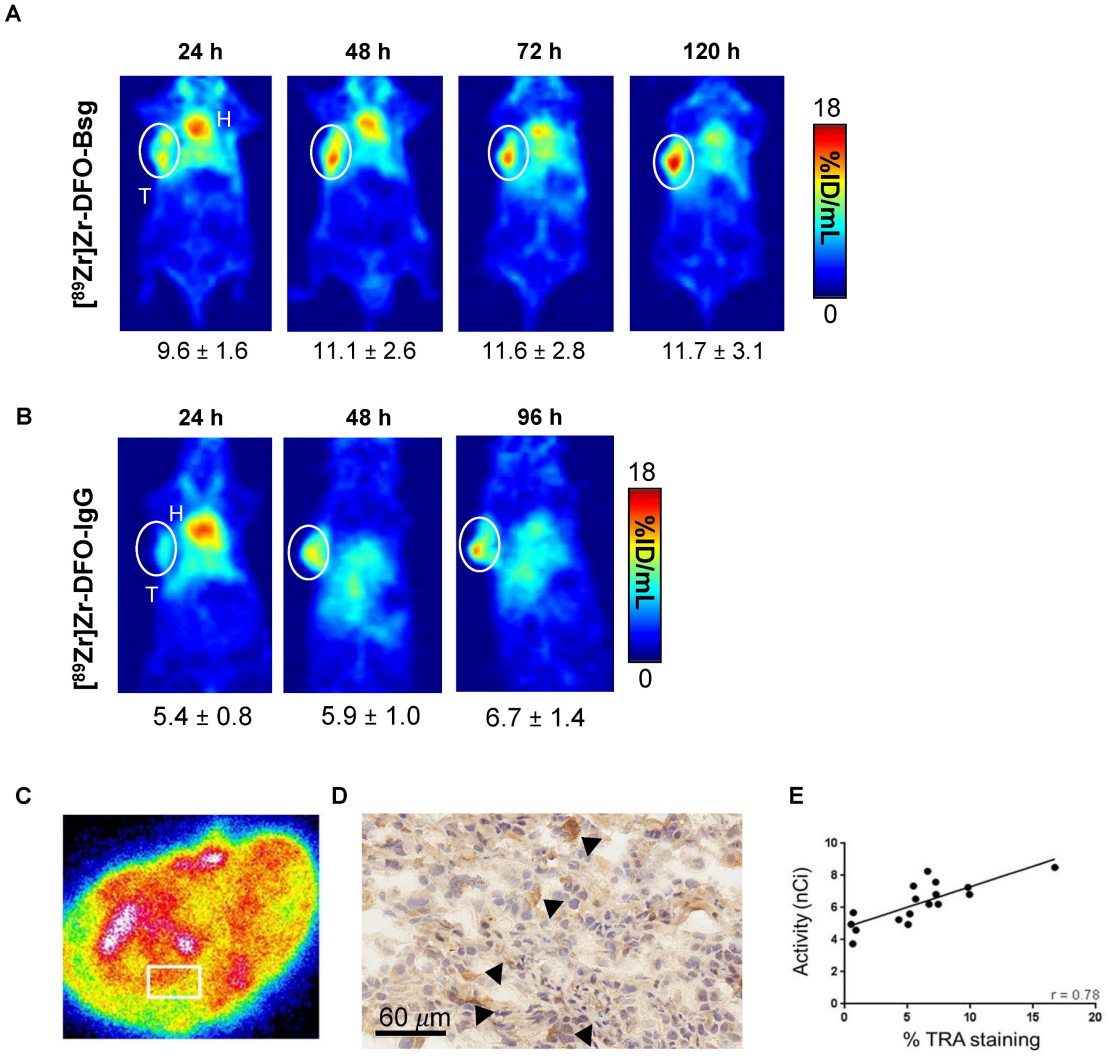
***In vivo* PET Imaging and *ex vivo* analysis. A.** PET images obtained with [^89^Zr]Zr-DFO-Bsg from 24 to 120 h p.i. demonstrates sustained tracer accumulation in DU-145 tumors (white circle, T=tumor, H=heart). **B.** Accumulation of [^89^Zr]Zr-DFO-IgG is lower than the TRA-specific probe in a separate cohort of mice. **C.** *Ex vivo* autoradiography of DU-145 exhibits focal heterogeneous localization of the tracer. The white box represents area where tracer uptake was measured. **D.** Immunohistochemistry of tumor sections (60 μm, 40x) display more intense brown staining (black arrows) indicating TRA expression. **E.** A Spearman correlation analysis comparing tracer uptake in the tumor (nCi) vs. %TRA-1-60 staining by IHC showed a positive correlation of r = 0.78.

To evaluate the potential of [^89^Zr]Zr-DFO-Bsg to identify TRA^+^ cells in established models of metastatic prostate cancer, we used DU-145, which exhibit high TRA expression compared to other PCa xenografts (e.g. 22rv1 and PC3) (**Figure S2A**). ImmunoPET imaging delineated DU-145 xenografts as early as 4 h p.i. with the radiotracer (**Figure 2A**). Tracer uptake peaked and appeared retained in the tumor between 24 and 120 h p.i. Non-specific IgG radiolabeled with Zr-89 showed low accumulation of the radiotracer in tumors (**Figure 2A-B**).

Autoradiographs of sectioned DU-145 tumors showed focal, heterogeneous localization of the TRA-specific tracer in different regions of the tumor (**Figure 2C**). Immunohistochemistry of a serial tumor section revealed TRA expression (**Figure 2D, Figure S2B**). Importantly, we observed a positive correlation between tracer binding and TRA expression, which confirmed the specificity of the tracer for the antigen (**Figure 2E**).

### Co-expression of TRA-1-60 with other cancer stem cell markers in DU-145 tumors

**Figure 3:**
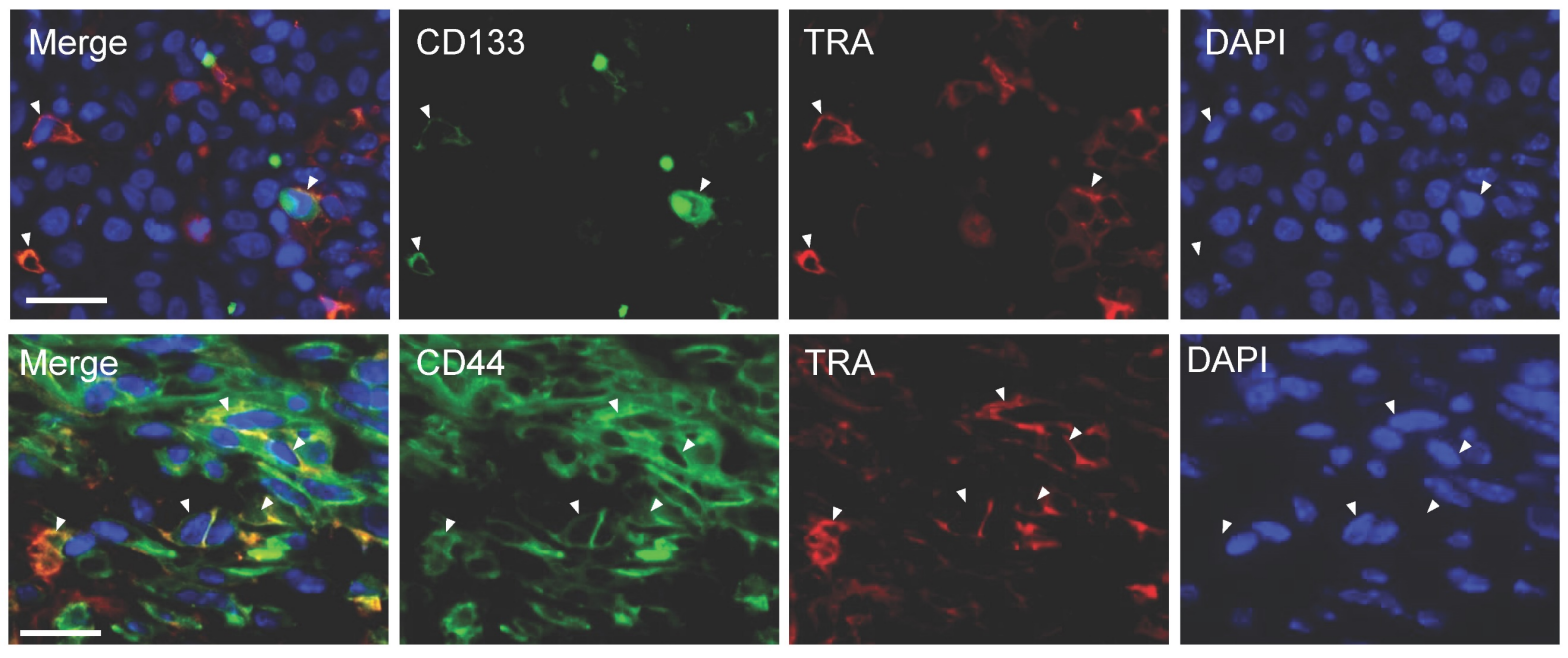
Co-expression of TRA-1-60 (TRA), CD133, and CD44 in DU145 tumor. Heterogeneous DU-145 tumor sections stained for either CD133 (green), CD44 (green) or TRA (red). DAPI (blue) was used to identifying nuclei. Representative cells both expressing CD44/TRA or CD133/TRA were marked with white arrowheads. Scale bar, 50 *µ*m.

We next examined whether other CSC markers like CD133 and CD44 are co-expressed with TRA in DU-145 tumor sections via immunofluorescent staining. DU-145 tumors showed abundant CD44 expression whereas minimal CD133 expression was displayed. Double staining displayed cells co-expressing both markers with TRA (**Figure 3**). However, not all cells show co-expression suggesting that TRA expression is highly regulated and may play a different role from these CSC markers. Moreover, there appears to be higher CD44/TRA positive staining than CD133/TRA.

### TRA-1-60 promotes tumor growth and expression correlates with tumor volume

**Figure 4:**
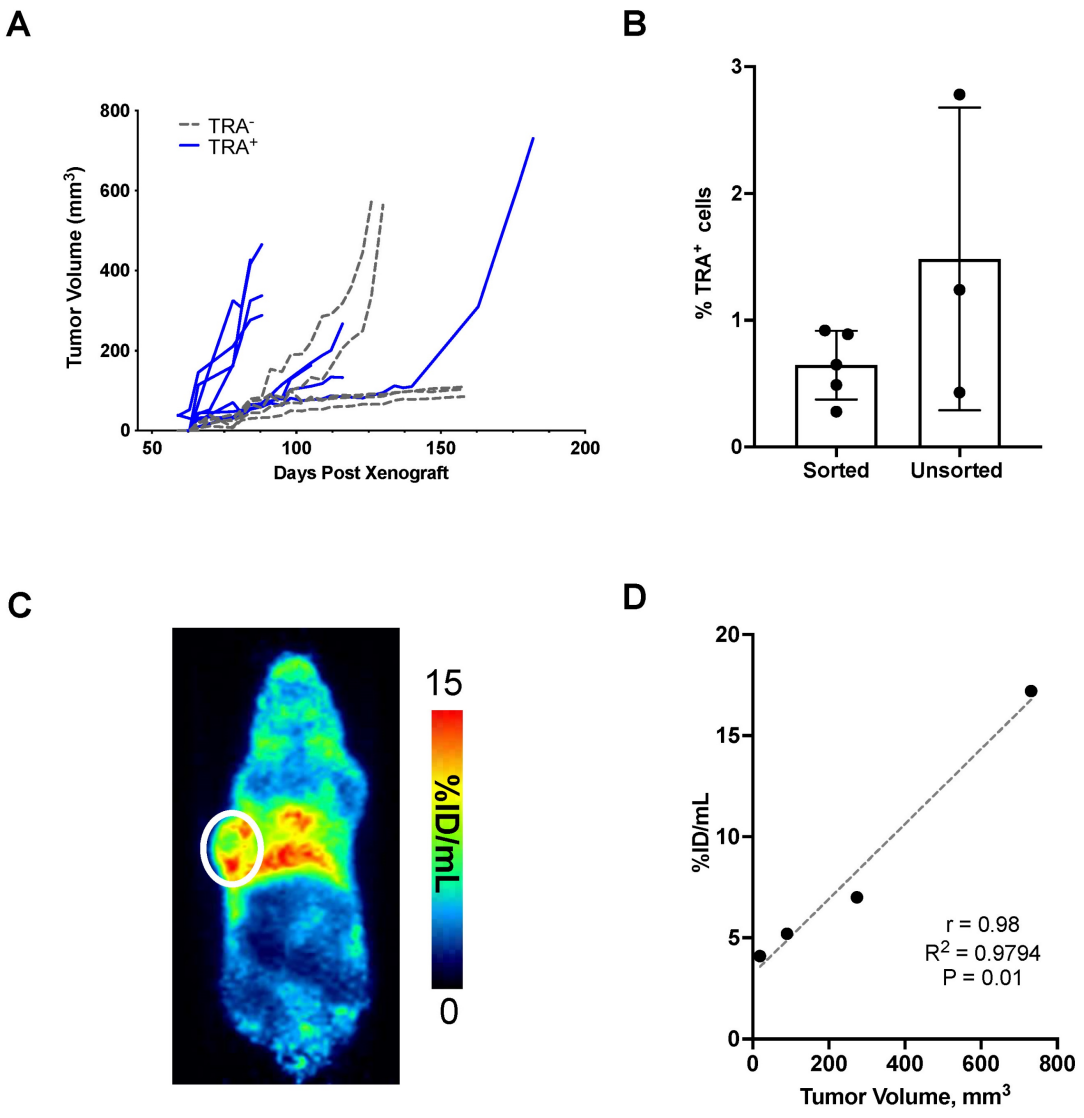
** Tumorigenicity of TRA^+^ cells. A.** TRA^+^ and TRA^-^ cells sorted from DU-145 tumors were re-implanted in mice. Tumors developed faster and at higher incidence with TRA^+^ enriched versus negative cells. **B.** *Ex vivo* analysis of %TRA^+^ cells from TRA^+^ sorted and heterogeneous xenografts. **C.** TRA^+^ xenografts imaged at 48 h p.i. of [^89^Zr]Zr-DFO-Bsg displayed tumor (white circle) accumulation **D.** A Spearman correlation of tracer uptake versus tumor volume in TRA^+^ sorted xenografts shows a strong positive correlation.

TRA^+^ and TRA^-^ cells were sorted and enriched from dissociated DU-145 tumors. Cells were subsequently re-implanted and tumor growth was monitored over time. Tumors developed in eight of the nine mice implanted with 2,500 TRA^+^ cells (**Figure 4A**). Two of the three mice that were seeded with lower TRA^+^ cells (~1,000 cells) developed into tumors (**Figure S3A**). In mice implanted with TRA^-^ cells, two out of seven mice grew tumors (>500 mm^3^) by 80 days post-xenograft. The rest demonstrated stunted growth, remaining at <100 mm^3^ at the end of the study, 158 days after seeding. Tumors were excised to evaluate the population of TRA^+^ cells in comparison to DU-145 xenografts that were not enriched for TRA. Flow cytometry analysis showed that there was no difference between the TRA^+^ and heterogeneous DU-145 xenografts. This suggests strict regulation of TRA expression, consistent with other reports that CSC cell surface markers (e.g., CD133) maintain a low frequency of expression and generate negative-expressing progeny despite enrichment (**Figure 3B**) 36. Indeed, our own cell sorting analysis showed very low population of cells that co-express both CD133 and TRA (**Table S1**); cells expressing CD133 alone are more abundant than those that are TRA^+^. TRA^-^ tumors were also evaluated for TRA expression by flow cytometry, however cell viability was low, hindering the analysis (**Figure S3B**). Tumor-draining lymph nodes (axillary) were also analyzed, however, no TRA^+^ cells were observed. *In vivo* imaging studies at 48 h p.i. of [^89^Zr]Zr-DFO-Bsg showed tumor uptake in enriched palpable TRA^+^ tumors (**Figure 3C-D**). Analysis of TRA^+^ tumor volumes vs. tumor uptake displayed a strong correlation (r = 0.98) (**Figure 3E**). Taken together, our findings suggest that TRA expression stimulates PCa tumorigenesis and that TRA-specific immunoPET can sensitively identify TRA^+^ cells in tumors.

### TRA-1-60-targeted radiopharmaceutical therapy (RPT) suppresses tumor growth and proliferative activity

Radiolabeling of [^177^Lu]Lu-CHX-A”-DTPA-Bsg was straightforward with radiolabeling yields of >87%. Pure product (>98%) was achieved after centrifugal filtration. The specific activity was 4.2 ± 0.1 mCi/mg. SPECT images obtained at 48 h (**Figure 4A**, left) and 120 h (**Figure 4A**, right) p.i. showed the accumulation of [^177^Lu]Lu-CHX-A”-DTPA-Bsg in DU-145 tumor. Tissue distribution at 120 h p.i. confirms low non-specific binding in most organs particularly in radiation sensitive tissues such as the kidneys and gastrointestinal organs (**Figure 4B**). Radioactivity in the blood persisted at 120 h p.i., implying that optimization of the tracer is necessary to improve pharmacokinetics.

DU-145-bearing mice were treated with 18.5 MBq (~500 mCi) of [^177^Lu]Lu-CHX-A”-DTPA-Bsg displayed tumor suppression (**Figure 4C**). A separate cohort of mice were administered higher activities at 27.8 MBq (~750 mCi) to examine if the outcomes could be improved. Tumor progression appeared blunted in the high versus low activity group at earlier time points but was not significantly different at the end of the study. Overall, tumor growth was suppressed in both treated groups compared to that in the untreated mice. To confirm that the tumor suppression was due to [^177^Lu]Lu-CHX-A”-DTPA-Bsg alone, a separate group of DU-145 tumor-bearing mice was injected with non-radiolabeled Bsg. No difference in tumor progression was observed between the Bsg-treated and untreated control groups (**Figure 4D**).

**Figure 5:**
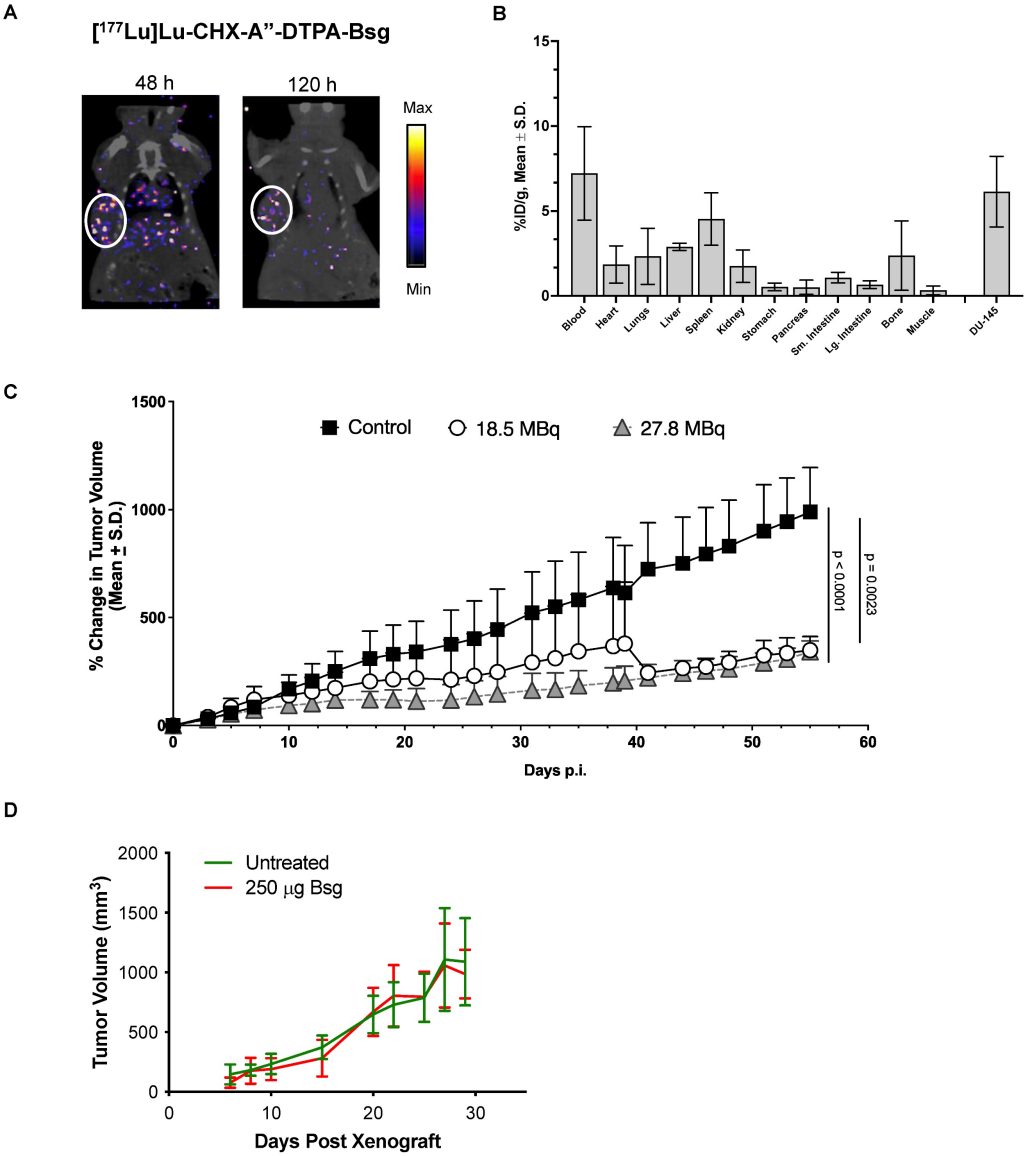
** Radiopharmaceutical therapy with** [^177^Lu]Lu-CHX-A”-DTPA-Bsg. **A**. SPECT/CT images display tumor accumulation with clearance from non-specific tissues after 120 h p.i. **B.** Tissue distribution at 120 h p.i. **C.** Tumor growth is suppressed in mice administered with high (27.8 MBq) and low (18.5 MBq) activities. **D.** Unmodified Bsg alone does not elicit therapeutic response.

Tumor sections were tested for expression of TRA after TRA RPT and for proliferative activity by immunohistochemical staining for Ki-67, a known nuclear marker of proliferation (**Figure 5A**) 37. Untreated tumors display elevated TRA expression whereas those that are treated demonstrate lower expression. Of note, expression of TRA appears diminished with increasing dose of the RPT agent. Cell proliferation appears to be also markedly decreased as evidenced by lower nuclear Ki-67 staining. Quantification of Ki-67 index (% of positive cells) displayed dose-dependent response, with tumors treated with the highest activity demonstrating the lowest proliferating cells (32.4 ± 8.5 %) versus control (88.0 ± 3.8 %, p <0.0001) (**Figure 5B**). Tumors administered with low activity RPT also exhibited decreased proliferation (50.2 ± 10.5 %, p = 0.001).

### Evaluation of radiotoxicity via clinical chemistry

**Figure 6:**
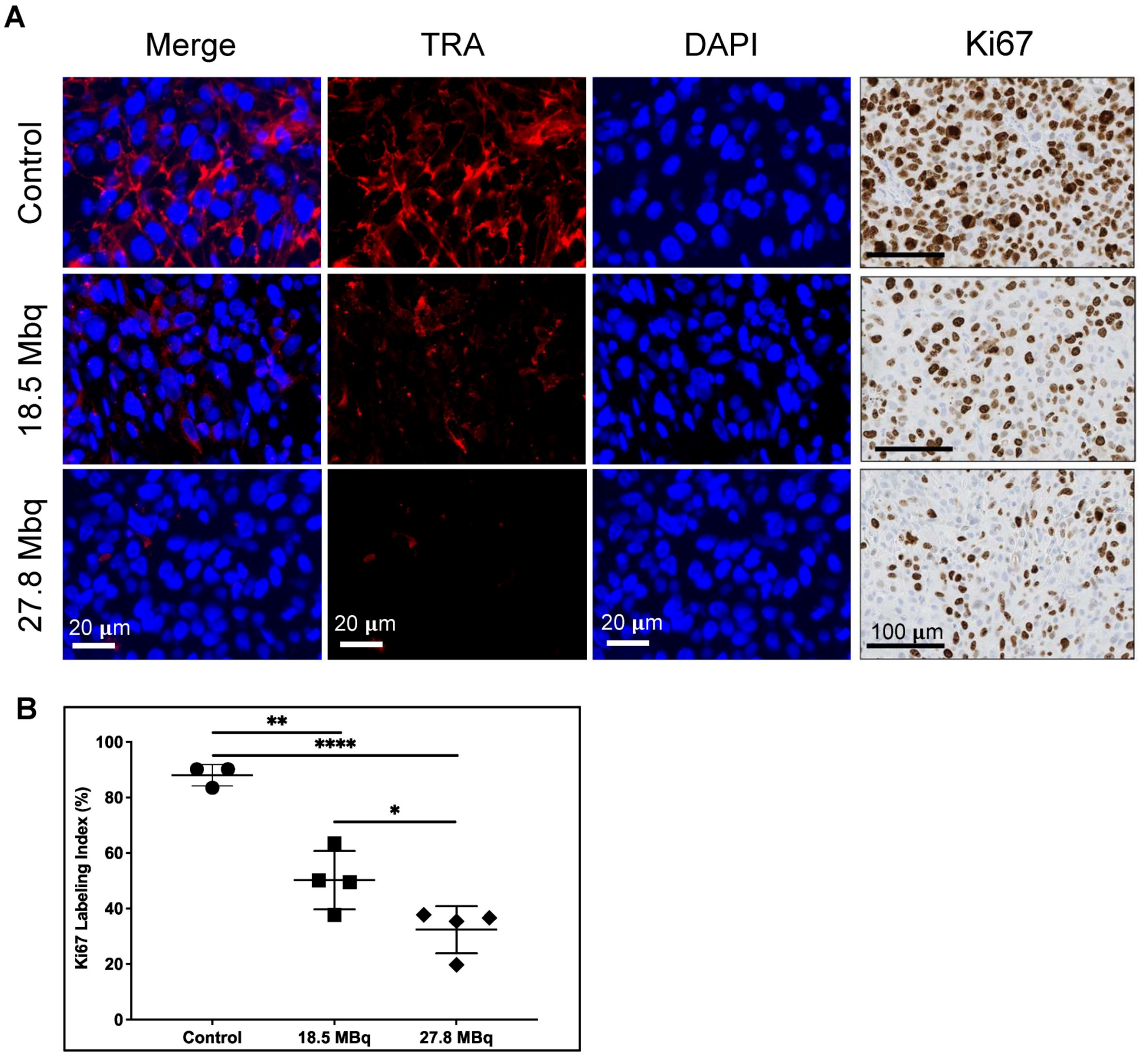
** TRA-1-60 (TRA) expression and proliferative activity of DU-145 tumors after treatment. A.** TRA expression (red) is diminished after treatment with 18.5 MBq (middle) and 27.8 MBq (bottom) activities of [^177^Lu]Lu-CHX-A”-DTPA-Bsg compared to control (top). Cell proliferation displayed via Ki-67 immunostaining (left panels) show decreased proliferative activity in treated tumors. **B.** Quantification of Ki67 marked decreased proliferation in both treated groups in a dose-dependent manner versus control untreated tumors. *P = 0.0425, **P = 0.0010, ****P<0.0001

Hematologic toxicity was examined at five days post-injection, when most of the RPT agent has accumulated in the tumor and ensured that Lu-177 had undergone almost a full half-life of decay. Testing was also conducted at the end of the study (60 d p.i.) to monitor for radiotoxicity after Lu-177 has completely decayed. No significant decrease in white blood cells (WBC) (**Figure 6A**) was observed in mice treated with the highest activity of the RPT agent compared with the control, suggesting that lymphopenia did not occur in the treated groups. No anemia was observed in the treated groups as shown by the comparable levels of red blood cells (RBC) (**Figure 6B**), hemoglobin (HGB) (**Figure 6C**) and hematocrit (HCT) (**Figure 6D**) with untreated mice across all time points. Platelet levels were also unaffected (**Figure 6E**). Blood urea nitrogen (BUN) was relatively stable and similar between control and at both timepoints for treated groups, indicating normal kidney function (**Figure 6F**). Liver activity was examined by measuring alanine aminotransferase (ALT) and alkaline phosphatase (ALP). Slightly elevated levels of ALT were observed in the treated groups but were not significantly different from the control (**Figure 6G**). Levels of ALP were elevated at five days p.i. with the RPT agent but were not found to be significantly different from controls (**Figure 6H**). At the end of the study, ALP values appeared to normalize. Weights of the rodents were monitored overtime starting from the day before dosing (**Figure 6I-K**). No weight loss was observed for the mice administered low levels of activity, however, 2 out of 4 mice showed >20% weight loss after 2 weeks of administering the agent, suggesting a subacute dose-dependent toxicity not detected within the five-day timeframe of our lab studies.

### Radiation effects of [^177^Lu]Lu-CHX-A”-DTPA-Bsg on liver and kidney histology

**Figure 7:**
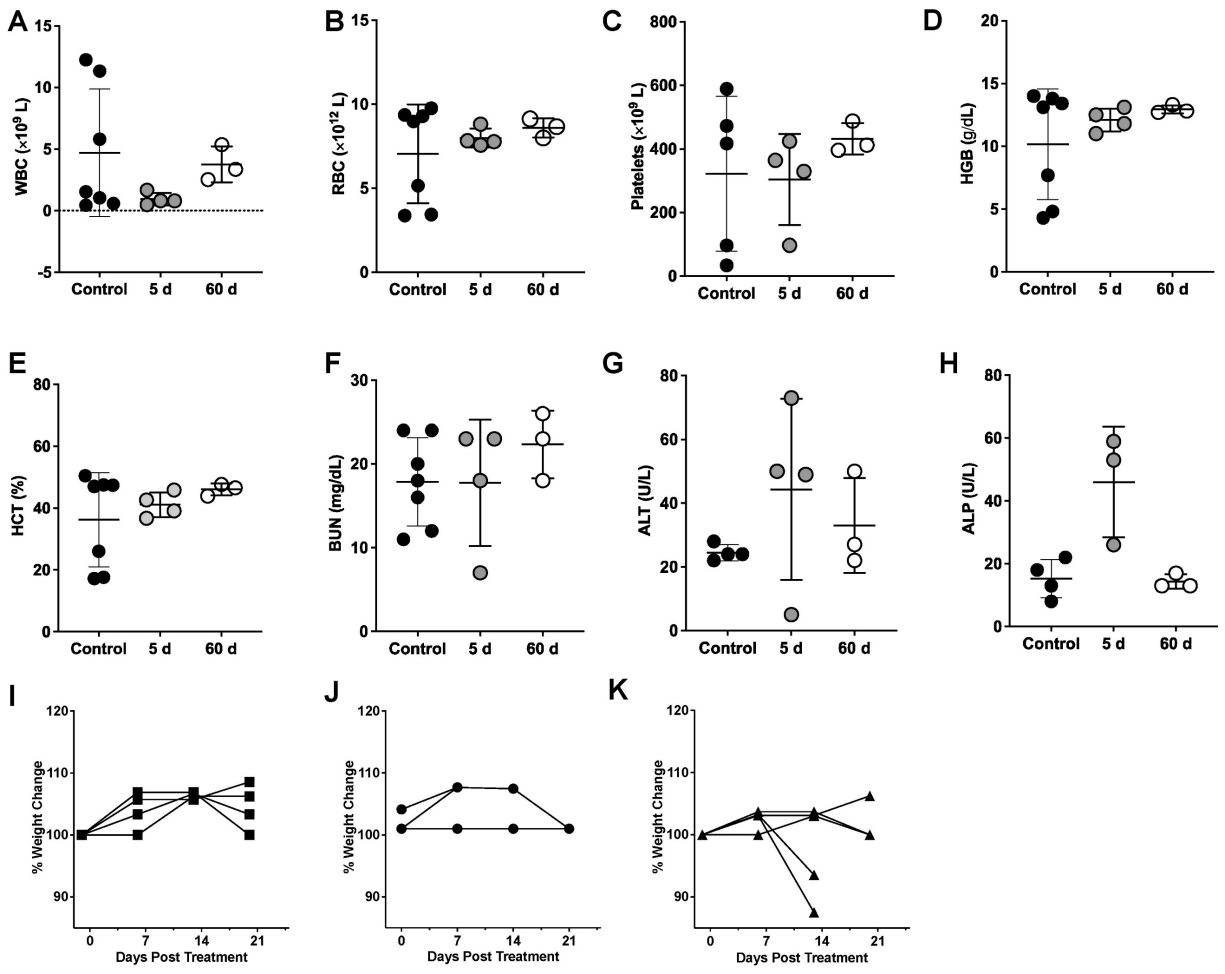
** Hematology (CBC) and serum chemistry analysis of DU-145 xenograft mice administered with high activity (27.8 MBq) [^177^Lu]Lu-CHX-A”-DTPA-Bsg. A.** WBC **B.** RBC **C.** platelets, **D.** hemoglobin (HGB), **E.** hematocrit (HCT), **F.** blood urea nitrogen (BUN), **G.** alanine aminotransferase (ALT), **H.** alkaline phosphatase (ALP). Assessment of % weight changes in **I.** control and treated mice at **J.** 18.5 MBq and **K.** 27.8 MBq.

Radiation therapy can damage the renal glomeruli, tubulo-interstitium and renal vasculature within the kidney, depending on the location and amount of radiation dose deposition 38. Some of the histopathological findings of radiation-induced renal injury include glomerular atrophy, glomerulosclerosis, mesangial proliferation, renal tubular dilatation and/or atrophy, interstitial inflammation, fibrosis and vascular thrombosis. Renal tissues were evaluated for radiation-induced damage using a previously reported scoring system 39. None of the renal samples demonstrated histological evidence of radiation-induced injury by routine light microscopy within the timeframe of this study (**Figure 7A-B**). Importantly, while late nephrotoxicity may not become apparent until 3-9 months after treatment with small-molecule RPTs, nephrotoxicity is uncommon for full-length antibody-based RPT 40.

A series of pathological changes can also be seen in the liver secondary to radiation therapy including centrilobular congestion, hepatocellular necrosis, venous obstruction, and fibrosis 41,42. Liver tissue was evaluated for radiation-induced damage using a previously reported scoring system 42. None of the liver samples demonstrated evidence of radiation-induced injury by routine light microscopy at the timeframe evaluated (**Figure 7C-D**). Most of the samples were histologically unremarkable. One liver sample showed remarkable for inflammatory changes including scattered microabscesses and microgranulomas (**Figure S4**). These types of inflammatory changes have a variety of etiologies (the most common being infectious disease) but are not typical for radiation-induced injury. Further investigation is required to determine the etiology in this mouse.

## Discussion

Here, we report the successful development of a theranostic strategy for imaging and selective ablation of TRA-expressing prostate cancer stem cells. To the best of our knowledge, this is the first radionuclide therapy agent developed to selectively target pluripotent cells *in vivo* with a favorable toxicity profile. To date, several RPT studies targeting CD133, a CSC marker of various malignancies have been conducted but have focused primarily on colorectal cancers 43,44. Previous reports have shown CD133 expression in PCa. However, its role remains controversial, with conflicting observations regarding its function as a PCa CSC marker 45. Notably, unpublished work from our lab has failed to show that CD133 promotes tumorigenesis in prostate cancer models. Our strategy centers on targeting TRA because of its key role in tumorigenesis which was empirically shown here by the growth of enriched TRA^+^ DU-145 cells as well as work by Rajasekhar *et al*. 29. Furthermore, its broad expression in many cancer phenotypes based on patient tissue IHC from our previous findings 33 expands the application of our agent. Using a radiotheranostic approach, we achieved sensitive detection of TRA^+^ CSCs via immunoPET, setting the stage for RPT ablation of these cells within the heterogeneous tumor mass. This essentially addresses the goal of eradicating the molecular drivers of tumor relapse and drug resistance.

[^177^Lu]Lu-CHX-A”-DTPA-Bsg elicited a therapeutic response in DU-145 metastatic PCa cell-derived xenograft model in a dose-dependent manner with the highest activity showing the highest inhibition of TRA expression. Despite the observed weight loss in the highest administered activity, we did not observe evidence of acute (5 d p.i.) and long-term (60 d) radiotoxicity, suggesting that the activities administered are safe and tolerated in mice. Tumor proliferation marked by Ki-67, a nuclear marker of active proliferation, confirmed anti-tumor effects, with proliferative activity significantly reduced in the treated groups compared to controls. Interestingly, the tumors treated with the highest activity had fewer proliferating cells compared to the low activity treatment groups, despite no differences in tumor volume at the end of the study. Unsurprisingly, this suggests a tumor dose-response correlating to the magnitude of radiation deposited within the tumor.

Notably, a complete response was not achieved, but the tumor progression was significantly inhibited in both treatment groups. This is likely due to the moderate TRA expression that limited the accumulated radiation dose within the tumor. Nevertheless, tumor suppression was still accomplished even with <3-5 % TRA^+^ cells present. Many studies have examined repeated or fractionated therapy to increase the therapeutic dose to tumors, increase efficacy and minimize off-target toxicities 35. Alternatively, the utility of alpha-emitting radionuclides can also be explored to further induce DNA damage. These options are feasible as TRA RPT is tolerated even at the highest dose administered. Moreover, we posit that TRA RPT combined with other tumor-responsive standard-of-care therapies and/or those that enhance DNA damage can lead to effective tumor debulking.

Approximately >25% of prostate cancer patients experience biochemical recurrence after radical prostatectomy with curative intent 46,47. To prevent the risk of metastatic progression, salvage radiation therapy to the prostate bed is typically deployed for such patients under the assumption that most recurrences are local 48. The distant spread of the disease limits therapeutic options to life-prolonging yet non-curative systemic therapy. While treatment for mCRPC include taxane-based chemotherapy, recent FDA approvals of radium-223 for the treatment of bone-limited mCRPC and [^177^Lu]Lu-PSMA-617 represent effective RPT options for this disease. Despite these advances, most patients with mCRPC succumb to the disease within 2 years 15,49, underscoring the fact that a cure remains elusive. That CSCs contribute to aggressive progression and drug resistance of PCa has now become widely accepted 9. Therapeutic strategies should then be directed toward these drug-resistant cell populations to prevent recurrence and refractory disease. Along these lines, TRA RPT falls within this treatment paradigm as it can potentially enhance outcomes by focusing on the cell population to which resistance and metastasis are attributed. This study serves as proof of principle that pluripotent CSCs can be selectively targeted and killed and even though they make up <5% of the tumor population, overall tumor growth is suppressed due to lower cellular proliferation.

Collectively, the limitations of the study lie in the fact that TRA is a new and relatively unexplored biomarker in PCa as well as in other cancer phenotypes. Marginal information on CNA expression as well as protein expression in both primary and mCRPC was mined from existing databases. In TCGA, data are limited to primary cancer with only one metastatic tumor available, while the rest are primary PCa. SU2C/PCF data has <12% (51/444) of mCRPC that was analyzed for *PODXL*. In the RPT study, we were unable to compare outcomes of TRA-specific versus non-specific delivery of Lu-177. However, we believe that TRA RPT likely has enhanced tumor suppression stemming from the higher deposition of activity based on the PET imaging data. Further studies are needed to explore the sub-acute, acute and chronic effects of radiation damage to unambiguously demonstrate the safety profile of TRA RPT. Additionally, expansion of the study to other metastatic PCa models to enhance our understanding of the role of TRA expression in treatment outcomes is warranted. Future studies will test the synergistic benefits of TRA RPT in combination with non-CSC standard-of-care drugs such as docetaxel and/or PSMA RPT.

In conclusion, we described the successful development and implementation of a radiotheranostic approach for imaging and treating TRA^+^ prostate CSCs. Ablation of TRA^+^ cells *in vivo* displayed promising therapeutic activity by suppressing tumor growth in a metastatic PCa model. Rationale combinations using TRA RPT and standard-of-care treatments may be able to achieve improved cures by selectively killing cells responsible for resistance and metastasis development.

## Supplementary Material

Supplementary figures and table.

## Figures and Tables

**Figure 8:**
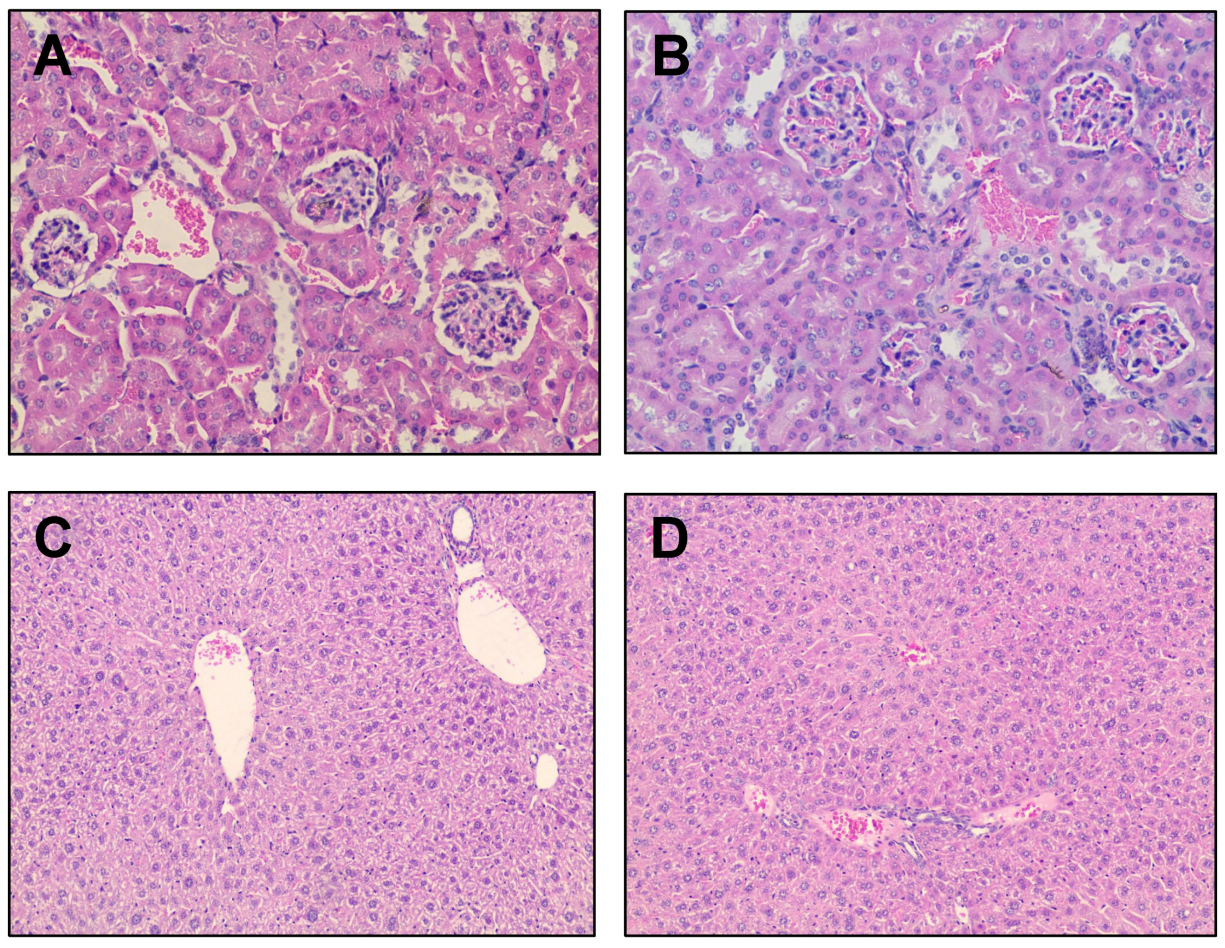
** Histologic evaluation via H&E**. Representative renal tissue from **A**. control and **B.** treated mice (H&E, 20×). Representative liver tissue from **C**. control and **D.** treated mice (H&E, 10×).
