# Supplementary material for: Selective ablation of TRA-1-60+ pluripotent stem cells suppresses tumor growth of prostate cancer
Source: Theranostics. 2023 Mar 27;13(7):2057–71. doi: 10.7150/thno.78915 (PMC10157726; doi:10.7150/thno.78915)
Supplement: Supplementary file 1 — Supplementary figures and table. [file thnov13p2057s1.pdf]

Supplementary Data

A

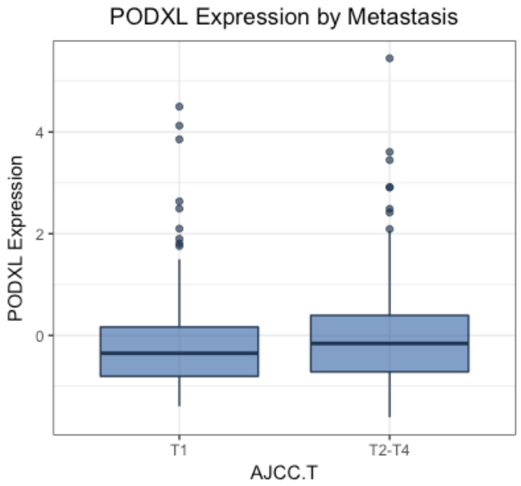

B

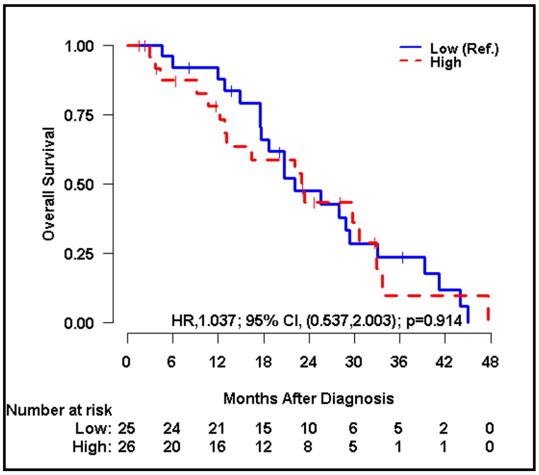

Figure S1 A. Larger tumors (T2-4) express higher PODXL expression. **B.** The Kaplan-Meier curve of overall survival (OS) by CNA levels of PODXL (High vs. Low, Low as reference) in metastatic prostate cancer. The CNA level was dichotomized into two groups (High vs. Low) by median. The p-value was obtained by a log-rank test. HR and CI stand for 'hazard ratio', and 'confidence interval', respectively.

A

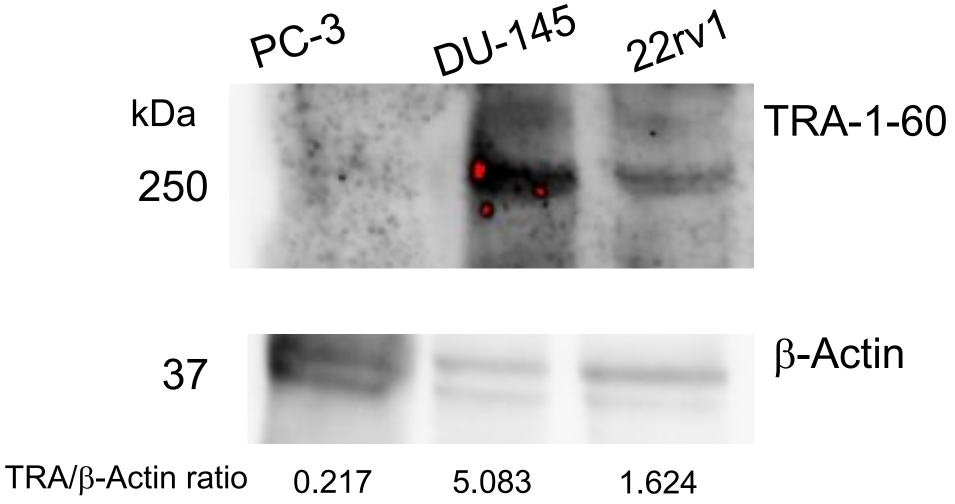

B

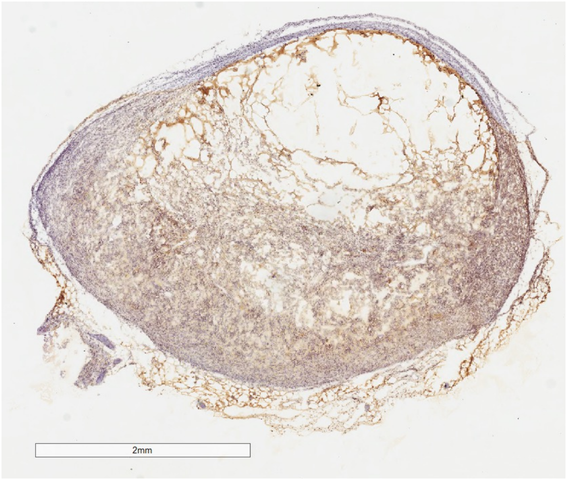

Figure S2 A. TRA-1-60 is expressed in prostate cancer xenografts in the following order: DU-145 > 22rv1 > PC-3. **B.** TRA-1-60 immunohistochemistry showing the full view of DU-145 tumor section.

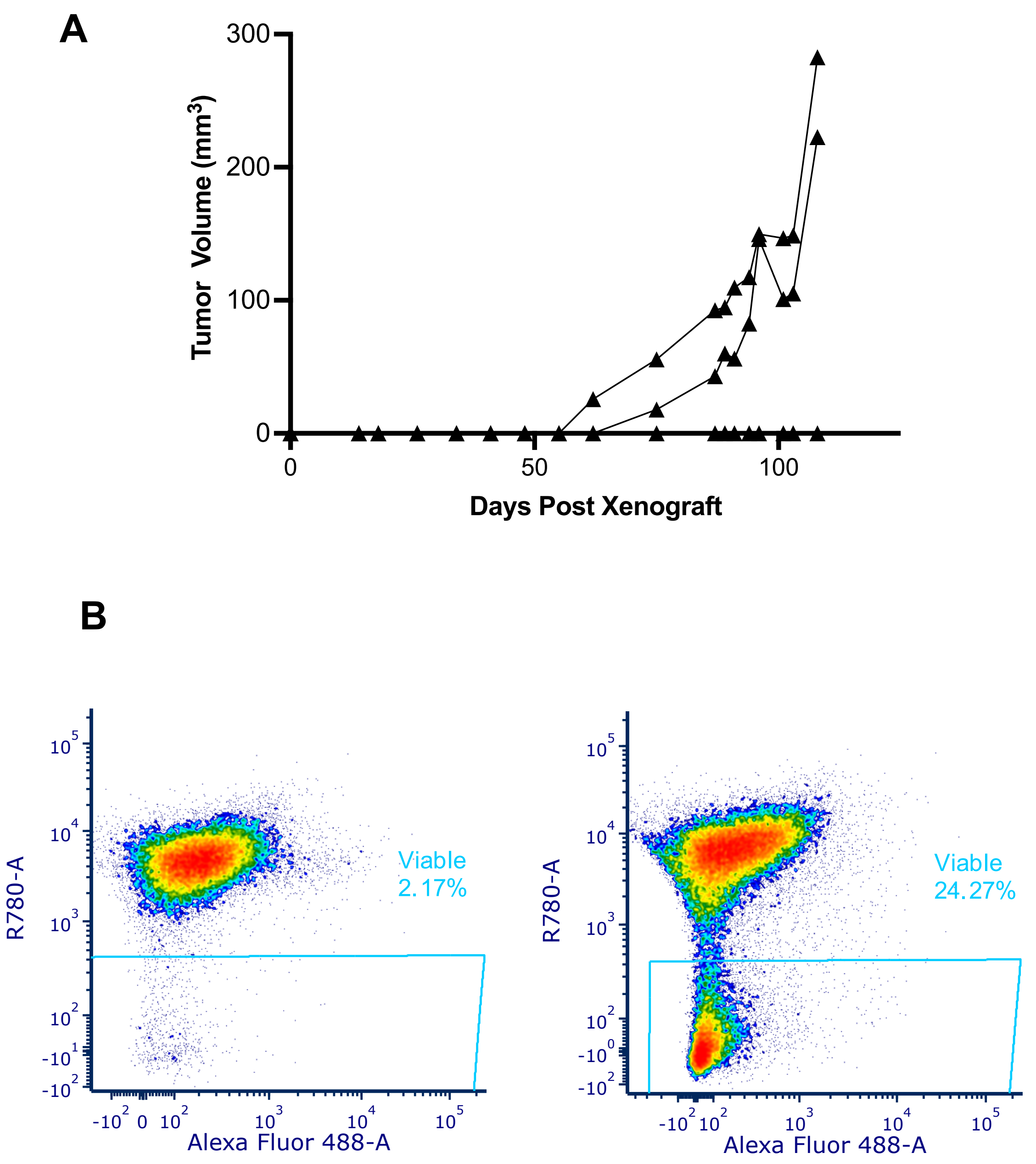

Figure S3. A. Tumors (2 out of 3) developed from 1,000 TRA$^+$ cells enriched from DU-145 cell-derived xenografts. **B.** Representative FACs viability plots of TRA$^-$ (left) and TRA$^+$ (right) cells dissociated from enriched tumors.

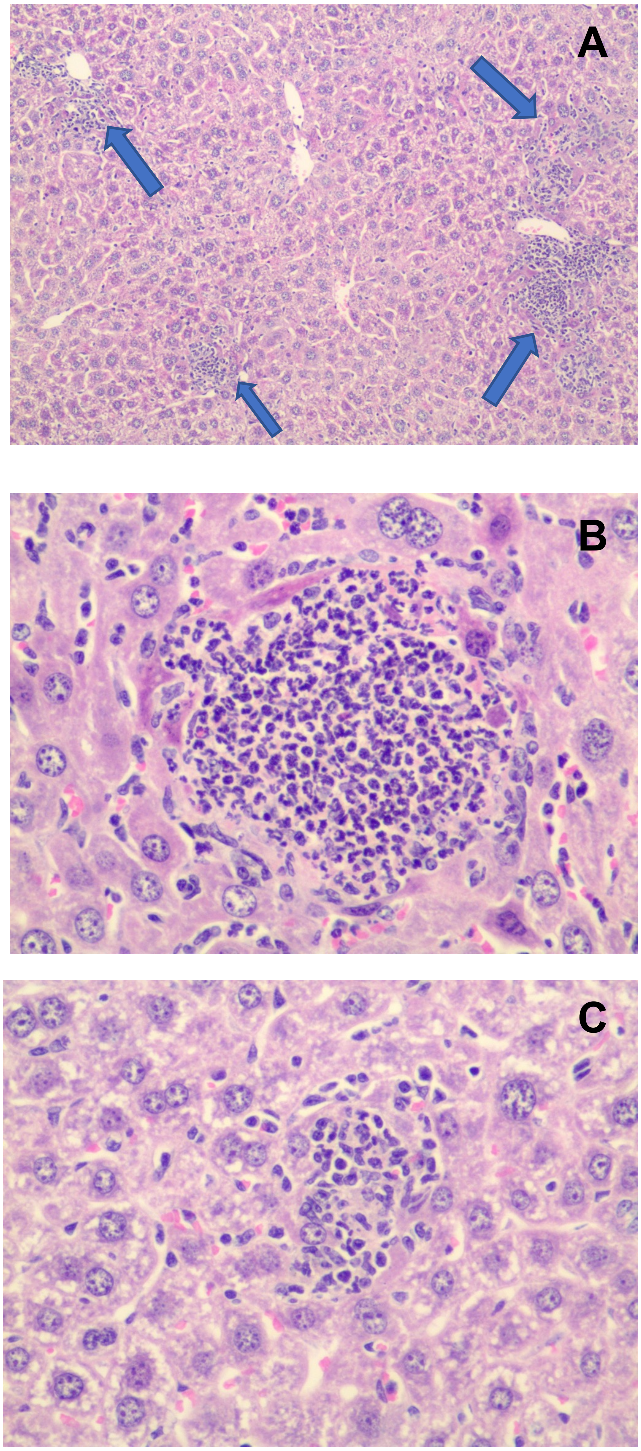

Figure S4 Inflammatory Changes Identified in Liver Samples from DU-145 mice. A. Scattered inflammatory changes (at arrows) were present throughout the hepatic parenchyma of mouse (H&E, 10×); B. High power view of hepatic microabscess (H&E, 40×); C. High power view of hepatic microgranuloma (H&E, 40×).

Table S1. Cell population analysis of DU-145 dissociated tumor via FACS.

Stain	% Cells	# of Cells Collected
$TRA^+/CD133^+$	0.11%	1,601
$TRA^+/CD133^-$	0.62%	10,433
$TRA^-/CD133^+$	11.4%	165,203
$TRA^-/CD133^-$	88%	1,362,644
